# Supplementary material for: Association of maternal, obstetric, fetal, and neonatal mortality outcomes with Lady Health Worker coverage from a cross-sectional survey of >10,000 households in Gilgit-Baltistan, Pakistan
Source: PLOS Glob Public Health. 2024 Feb 27;4(2):e0002693. doi: 10.1371/journal.pgph.0002693 (PMC10898742; doi:10.1371/journal.pgph.0002693)
Supplement: S1 Checklist — PLOS inclusivity in global research checklist. (DOCX) [file pgph.0002693.s001.docx]

Inclusivity in global research

PLOS’ policy on inclusivity in global research aims to improve transparency in the reporting of research performed outside of researchers’ own country or community and ensures that PLOS publications reporting global research adhere to high standards for research ethics and authorship. Authors of relevant research articles may be asked to complete the questionnaire below, which outlines ethical, cultural, and scientific considerations specific to inclusivity in global research. This questionnaire may be requested when researchers have travelled to a different country to conduct research, if research uses samples collected in another country, research with Indigenous populations or their lands, or if research is on cultural artefacts. Researchers travelling to another country solely to use laboratory equipment will not normally be required to complete the questionnaire. However, the questionnaire can be requested at the journal’s discretion for any submission – if you have been requested to complete this questionnaire by the PLOS journal you submitted to, please do so.

Please complete the questionnaire below and include this as a Supporting Information file with your manuscript. Note that if your paper is accepted for publication, this checklist will be published with your article in the supporting information files. Please ensure that you reference the checklist in the main body of your manuscript. We suggest adding a subsection ‘Inclusivity in global research’ to your Methods section and adding the following sentence: “Additional information regarding the ethical, cultural, and scientific considerations specific to inclusivity in global research is included in the Supporting Information (SX Checklist)”

The questions have been designed to be applicable to a wide range of study types, and there are subsections for both human subjects research and non-human subjects research. If any of the questions are not relevant to your research please mark them as “N/A” as appropriate.

**Ethical considerations, permits and authorship**

*This section is applicable to all research types.*

Provide details as to who granted permissions and/or consent for the study to take place in the Methods section of your manuscript. This should include the names of **all** ethics boards, governmental organizations, community leaders or other bodies that provided approval for the study. If individuals provided approval refer to these people by their role or title but do not list their name(s).

**Reported on page number:** Details regarding ethics board approvals are described on page 7 of the revised manuscript. This includes mention of ethics board approval at The Hospital for Sick Children (REB 1000063672) and Aga Khan University (ERC 5250), as well as governmental approval from the National Bioethics Committee of Pakistan (NBC 580). In the revised manuscript (lines 128–132), we have also included details regarding letters of cooperation following discussion with community leaders (described below), which were signed by the Gilgit-Baltistan Secretary of Health.

If there were any deviations from the study protocol after approval was obtained please provide details of these changes in the Methods section of your manuscript.

**Reported on page number:** Not applicable, no deviations from the study protocol occurred in the study.

Did this study involve local collaborators that are residents of the country where the research was conducted or members of the community studied? If you do not have any authors from said communities, please provide an explanation for this below.

This study was conducted as part of a collaboration between The Hospital for Sick Children (Toronto, Canada), Aga Khan Health Services, Pakistan (Gilgit and Karachi, Pakistan), and Aga Khan University (Karachi, Pakistan). Several co-authors are current residents of Pakistan (MY, SHK, IA, MK, FM, SP, MAK, and SBS), including several who reside in Gilgit-Baltistan (the region under study; MY, SHK, and MAK). These authors were crucial team members, and were involved in study design, implementation and oversight of field-based activities, and developing relationships with local government health officials to ensure their support for the cross-sectional survey and associated cluster randomized controlled trial.

Everyone listed as an author should meet PLOS’ criteria for authorship and all individuals who meet these criteria should be included in the author byline, rather than the acknowledgements. For further information please see the journal’s Authorship Policy.

**Human subjects research (e.g. health research, medical research, cross-cultural psychology)**

Did you obtain written informed consent from a representative of the local community or region before the research took place? How did you establish who speaks for the community? Details of written informed consent obtained from study participants should be reported separately in the Methods section of your manuscript.

During the study design phase (i.e. prior to conducting any field research), our study team met with representatives of the Gilgit-Baltistan Department of Health to discuss the study aims and proposed research activities. The discussions covered both the cluster randomized controlled trial as well as baseline survey from which this analysis was conducted. Letters of Cooperation were then signed by the Gilgit-Baltistan Secretary of Health following unanimous approval by review committee members. These documents mandated coordination with district health officers and the Lady Health Worker program, in alignment with Gilgit-Baltistan government policies.

Furthermore, to ensure community engagement, the study team engaged in informal meetings and discussions with community members, religious leaders, and ‘lambardars’ (influential representatives of certain populations within the community) to foster support and understanding for the study. Notably, it was not deemed necessary by our academic research ethics boards to obtain written informed consent from stakeholders involved in these informal meetings to proceed with the research activities.

How did members of the local community provide input on the aims of the research investigation, its methodology, and its anticipated outcome(s)?

Early in the study design phase, discussions with the Gilgit-Baltistan Department of Health were used to inform and guide our study aims to ensure they aligned with local health priorities. Through continued discussions with the local community members described above, we routinely incorporated feedback into the planned research activities. Examples of this feedback includes alterations to the planned study catchment area, composition of data collection team members (both female and male members), and respectful approaches to eligible households for enrollment and data collection. The submitted publication also resulted from discussions between Pakistani and Canadian representatives, expanding the results from an internal report to inform planned trial activities into a dedicated manuscript to disseminate findings of the maternal and newborn outcomes under study.

When engaging with the local community, how did you ensure that the informed consent documents and other materials could be understood by local stakeholders?

During the study design phase, engagement with local stakeholders (e.g., Gilgit-Baltistan Department of Health, community and religious leaders) was conducted by Pakistani members of the research team, sometimes jointly with Canadian research team members. Representatives of the Department of Health are trained experts in the medical field, with extensive knowledge of the study population and its health outcomes and priorities. These stakeholders therefore had a high degree of understanding regarding our study aims and proposed research activities, which was taken into consideration when executing Letters of Cooperation. In discussions with other community members (i.e., non-medical experts), our team made great effort to describe the rationale for the study and its proposed activities. This included the need for a randomized design during the intervention period, the need to collect representative baseline data prior to the intervention period (i.e., to understand the burden of maternal and newborn outcomes in the study catchment area), the specific research activities that would be used to collect these data, and how the team planned to report and disseminate findings from the study. These discussions were held in Urdu, the primary language in the region.

During data collection, teams of four study workers visited selected villages to administer the study questionnaire. All data collection forms were printed in Urdu, the predominant language in Gilgit-Baltistan. However, at least one member of each data collection team was also proficient in local languages specific to each region of the study, including Balti (spoken in Baltistan), Shina (spoken in Astore), and Brushiski (spoken in Nagar). Ensuring the presence of at least one team member fluent in the indigenous language was a top priority. Consenting research participants were also encouraged to ask questions during the consent discussion and data collection.

Will the findings of the research be made available in an understandable format to stakeholders in the community where the study was conducted (e.g. via a presentation, summary report, copies of publications, etc.)? Please provide details of how this will be achieved.

Yes, findings of the research will be made available to community stakeholders. In September 2022, Canadian members of the study team conducted field visits in Gilgit-Baltistan to share interim findings of this analysis to Pakistani research team members. During these discussions, we highlighted the need for presentations to the Gilgit-Baltistan Department of Health, district health offices, and the Lady Health Worker program. We also plan to conduct workshops to disseminate the study findings in an understandable manner to interested community members. In addition, we have included an Urdu version of the study abstract along with our submission.

**Non-human subjects research using specimens/ animals collected as part of the study, or those housed in archival collections. Examples include archaeology, paleontology, botany and zoology.**

Did the permission you obtained from a local authority to perform the study include an agreement on access to outputs and benefit sharing? This may include procedures to enable fair distribution of the benefits and resources arising from the research performed. Please include any details of Prior Informed Consent and Benefit Sharing Agreements obtained. These may be required by field-specific regulations, for example the Convention on Biological Diversity (CBD) and the associated Nagoya Protocol.

Not applicable, this study was conducted among human participants and households only.

If the material used in your study was imported, please A) provide the year it was imported and B) indicate whether permits were obtained to import/export the materials used, C) provide details of any permits obtained. If this information is not available, please indicate this.

Not applicable, this study was conducted among human participants and households only.

If you used archival specimens, please state how the material used in your study was acquired by the institute it is held in and provide details of any permits obtained for the original excavations/ sample collection. If this information is not available, please indicate this.

Not applicable, this study was conducted among human participants and households only.

How was the potential cultural significance of the materials collected in your study to local communities considered in your research design? Were Indigenous peoples and/or local researchers and institutions involved with archaeological excavations / collection of specimens? If so, please provide a description of their involvement.

Not applicable, this study was conducted among human participants and households only.

If your manuscript includes photographs of human remains please indicate whether authors obtained permission from descendants or affiliated cultural communities to do so.

Not applicable, this study was conducted among human participants and households only.
