# Supplementary material for: Association of maternal, obstetric, fetal, and neonatal mortality outcomes with Lady Health Worker coverage from a cross-sectional survey of >10,000 households in Gilgit-Baltistan, Pakistan
Source: PLOS Glob Public Health. 2024 Feb 27;4(2):e0002693. doi: 10.1371/journal.pgph.0002693 (PMC10898742; doi:10.1371/journal.pgph.0002693)
Supplement: S1 File — Alternative language (Urdu) abstract. (PDF) [file pgph.0002693.s002.pdf]

# گلگت بلتستان، پاکستان میں 10,000 سے زیادہ گھرانوں پر مشتمل کراس سیکشنل سروے کے ذریعے حمل اور دوران زچگی خاتون، جنین، اور نوزائیدہ اموات کے نتائج کا لیڈی ہیلتھ ورکرز کی کوریج کے تعلق پر مبنی جائزہ۔


**خلاصہ:**

پاکستان عالمی سطح پر زچگی، قبل از پیدائش دیکھ بھال اور پیدائش کے بعد نوزائیدہ بچوں کی اموات کی بلند ترین شرح والے ممالک میں شامل ہے۔ ان میں سے بہت سی اموات کو کم لاگت پر مشتمل وسیع پیمانے پر فراہم کردہ اقدامات (interventions) کے ذریعے ممکنہ طور پر روکا جاسکتا ہے جو کمیونٹی پر مبنی ہیلتھ ورکر پروگراموں کے ذریعے انتہائی دور دراز علاقوں میں رہنے والے خاندانوں تک پہنچائے جاسکتے ہیں۔ ہم نے جون سے اگست 2021 کے دوران گلگت بلتستان میں کلسٹر رینڈ امائزڈ کنٹرول ٹرائل (cRCT) کے آغاز میں 10,264 گھرانوں کا (Cross Sectional) سروے کیا تھا۔ اس سروے کا انعقاد مطالعہ کی جغرافیائی حدود کے اندر (Stratified, two-stage sampling design) یعنی آبادی کے لحاظ سے دو ذیلی گروپس سے لوگوں کو منتخب کر کے کیا گیا تھا جس کا بنیادی مقصد اس سروے میں شامل علاقوں میں نوزائیدہ بچوں کی اموات کی شرح کا تخمینہ لگانا اور کلسٹر رینڈ امائزڈ کنٹرول ٹرائل (cRCT) کے بارے میں معلومات فراہم کرنا تھا۔ اس مطالعے میں حاصل ہونے والے نتائج جن میں نوزائیدہ بچوں کی اموات، مردہ پیدائش، صحت کی سہولت پر زچگی، زچگی کے دوران ہونیوالی اموات، بعد از پیدائش خون کا زیادہ اخراج (postpartum hemorrhage)، اور ان علاقوں میں لیڈی ہیلتھ ورکر (LHW) کی کوریج شامل تھی۔ مطالعے کے اعداد و شمار (تناسب اور شرح) کو سیمپلنگ ڈیزائن کے مطابق جانچا گیا تھا، اور LHW کوریج اور زچگی / نوزائیدہ نتائج کے درمیان تعلق کو تلاش کرنے کے لیے (Mixed-effects Poisson Regression) جس میں (تجزیے کی ایک عمومی لکیری شکل جو اعداد و شمار اور ہنگامی جدولوں کے ماڈل کے لیے استعمال ہوتی ہے) کا انعقاد کیا گیا تھا۔ ہم نے 7,600 ایسی خواتین کی نشاندہی کی جن کے ہاں گزشتہ پانچ سالوں کے دوران بچوں کی پیدائش ہوئی تھی اور ان میں سے 13 فیصد خواتین کو بعد از پیدائش خون کے زیادہ بہاؤ / اخراج کا سامنا کرنا پڑا تھا۔ زچگی کے دوران اموات کی شرح کا تناسب 225 اموات فی 100،000 زندہ پیدائشوں میں (95% Confidence Interval [CI] 137–369) تھا۔ 12,376 کُل پیدائشوں میں سے، مردہ پیدا ہونے والے بچوں کی شرح 41.4 فی 1,000 پیدائشیں (95% CI 36.8–46.7) تھی اور پیدائش کے دوران ہونے والی اموات کی شرح 53.0 فی 1,000 پیدائشیں (95% CI 47.6–59.0) تھی۔ 11,863 زندہ پیدا ہونے والے بچوں میں اموات کی شرح 16.2 فی 1000 زندہ پیدائش تھی (95% CI 13.6–19.3) جبکہ ان میں سے 65 فیصد کی پیدائش (Health Facility) صحت کی سہولت پر ہوئی تھی۔ لیڈی ہیلتھ ورکر کے گھریلو دورے پیدائش کے بعد خون کے زیادہ اخراج (postpartum hemorrhage) اور پیدائش کے 7 سے 28 دن کے اندر ہونے والی بچوں کی اموات میں کمی لانے کا سبب بنے تھے۔ (ہر اضافی دورے پر خطرے کا تناسب [RR] 0.89 ، 95% CI 0.83–0.96) تھا جبکہ 28 دن کے اندر ہونے والی نوزائیدہ اموات کا تناسب (RR 0.80, 95% CI 0.67–0.97) تھا جس میں کمی واقع ہوئی ہے۔ مستقبل کے تحقیقی مطالعہ کی منصوبہ بندی سے آگاہ کرنے کے لیے (Intracluster correlation coefficients)، (کلسٹر ڈیٹا کی وابستگی کا ایک پیمانہ ایک ہی گروپ میں موجود اشیاء جو دوسرے گروپوں کے مقابلے میں ایک دوسرے سے زیادہ ملتی جلتی ہوں) کا بھی تخمینہ لگایا گیا تھا۔ گلگت بلتستان میں زچگی، زچگی کے دوران ہونے والی اموات اور پیدائش کے 28 دن کے اندر ہونے والی اموات کی بلند شرح 2030 کے پائیدار ترقی کے اہداف (Sustainable Development Goals) سے دور ہیں۔
