# Supplementary figures and images for: Association of maternal, obstetric, fetal, and neonatal mortality outcomes with Lady Health Worker coverage from a cross-sectional survey of >10,000 households in Gilgit-Baltistan, Pakistan
Source: PLOS Glob Public Health. 2024 Feb 27;4(2):e0002693. doi: 10.1371/journal.pgph.0002693 (PMC10898742; doi:10.1371/journal.pgph.0002693)

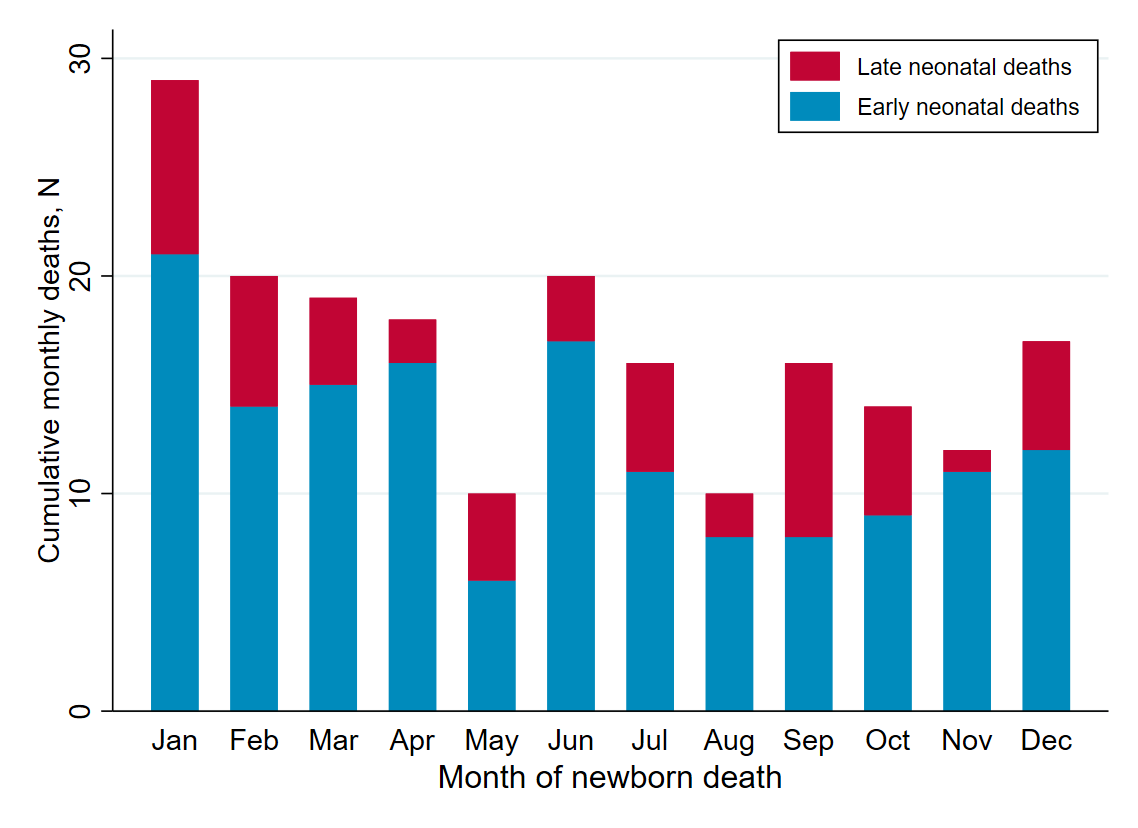


**S1 Figure**. Seasonality of cumulative monthly newborn deaths from July 2016–August 2021.

Supplement: S1 Fig — (DOCX) [file pgph.0002693.s003.docx]
