# Supplementary material for: Association of maternal, obstetric, fetal, and neonatal mortality outcomes with Lady Health Worker coverage from a cross-sectional survey of >10,000 households in Gilgit-Baltistan, Pakistan
Source: PLOS Glob Public Health. 2024 Feb 27;4(2):e0002693. doi: 10.1371/journal.pgph.0002693 (PMC10898742; doi:10.1371/journal.pgph.0002693)
Supplement: S1 Table — (DOCX) [file pgph.0002693.s004.docx]

| **S1 Table.** Newborn outcomes by place of birth. | | | | |
| --- | --- | --- | --- | --- |
| **Indicator** | **Place of birth^1^** | | **Risk ratio**  **(95% CI)** | ***P* value** |
|  | **Home/Other** | **Health facility** |  |  |
| Neonatal deaths, n / N (%) | 78 / 4692 (1.5) | 125 / 6914 (1.7) | --- | --- |
| Neonatal mortality rate (95% CI), per 1000 live births | 14.6 (10.7–19.8) | 17.1 (13.4–21.7) | 1.14 (0.76–1.72) | 0.528 |
| Early neonatal deaths, n (%) | 56 / 4757 (1.1) | 94 / 7059 (1.3) | --- | --- |
| Early neonatal mortality rate (95% CI), per 1000 live births | 10.7 (7.6–15.0) | 13.2 (10.0–17.4) | 1.22 (0.77–1.94) | 0.403 |
| Late neonatal deaths, n (%) | 22 / 4636 (0.4) | 31 / 6820 (0.4) | --- | --- |
| Late neonatal mortality rate (95% CI), per 1000 live births^2^ | 3.8 (2.1–7.0) | 3.7 (2.3–5.8) | 1.01 (0.47–2.16) | 0.985 |
| ^1^Not reported for 47 newborns. ^2^Denominator excludes live births who died in the first week of life, and live newborns who had not yet reached day 28 of life. | | | | |
