# Supplementary material for: Association of maternal, obstetric, fetal, and neonatal mortality outcomes with Lady Health Worker coverage from a cross-sectional survey of >10,000 households in Gilgit-Baltistan, Pakistan
Source: PLOS Glob Public Health. 2024 Feb 27;4(2):e0002693. doi: 10.1371/journal.pgph.0002693 (PMC10898742; doi:10.1371/journal.pgph.0002693)
Supplement: S2 Table — (DOCX) [file pgph.0002693.s005.docx]

| **S2 Table.** Non-parametric (Spearman) correlations between study indicators. | | | | | | | | | |
| --- | --- | --- | --- | --- | --- | --- | --- | --- | --- |
| **Indicator** | (1) | (2) | (3) | (4) | (5) | (6) | (7) | (8) | (9) |
| (1) Postpartum hemorrhage | --- | 0.26* | 0.04 | 0.21 | 0.15 | 0.29* | 0.35* | 0.34* | 0.17 |
| (2) Maternal death |  | --- | -0.01 | -0.09 | 0.22 | 0.28* | 0.15 | 0.19 | -0.01 |
| (3) Crude birth rate, 2020 |  |  | --- | -0.1 | -0.05 | 0.01 | 0.05 | 0.12 | -0.12 |
| (4) Health facility delivery^1^ |  |  |  | --- | -0.19 | -0.21 | -0.08 | -0.04 | -0.08 |
| (5) Stillbirth rate |  |  |  |  | --- | NC | 0.1 | 0.1 | 0.01 |
| (6) Perinatal mortality rate |  |  |  |  |  | --- | NC | NC | 0.09 |
| (7) Neonatal mortality rate |  |  |  |  |  |  | --- | NC | NC |
| (8) Early neonatal mortality rate |  |  |  |  |  |  |  | --- | 0.17 |
| (9) Late neonatal mortality rate |  |  |  |  |  |  |  |  | --- |
| NC=Not Calculated (i.e. not calculated because numerators are not mutually exclusive; e.g. stillbirths also included in perinatal death, early and late neonatal deaths included in all neonatal deaths). Values are Spearman’s rank correlation coefficient, and * indicates a statistically significant correlation (p<0.05). ^1^Among live births only. | | | | | | | | | |
