# Supplementary material for: Association of maternal, obstetric, fetal, and neonatal mortality outcomes with Lady Health Worker coverage from a cross-sectional survey of >10,000 households in Gilgit-Baltistan, Pakistan
Source: PLOS Glob Public Health. 2024 Feb 27;4(2):e0002693. doi: 10.1371/journal.pgph.0002693 (PMC10898742; doi:10.1371/journal.pgph.0002693)
Supplement: Attachment — Submitted filename: iNCK_MaternalNewborn_ResponseToReviewers.docx [file pgph.0002693.s006.docx]

November 13, 2023

Dear Editors,

Thank you for reviewing our manuscript entitled “Association of maternal, obstetric, fetal, and neonatal mortality outcomes with Lady Health Worker coverage from a cross-sectional survey of >10,000 households in Gilgit-Baltistan, Pakistan”. We welcome the opportunity to submit a revised manuscript for consideration of publication in *PLOS Global Public Health*.

We have carefully considered the feedback raised by the journal and peer reviewer. We have responded to each of these comments below, and where necessary have included line numbers which refer to track changes in the revised manuscript (tracked changes version) which has been provided in this submission. We believe that the paper has been strengthened by these revisions and are grateful to all reviewers for their time and contributions.

Thank you again for your consideration.

Sincerely,

Daniel S. Farrar & Dr. Shaun K. Morris

Daniel S. Farrar ([daniel.farrar@sickkids.ca](mailto:daniel.farrar@sickkids.ca))

Centre for Global Child Health, The Hospital for Sick Children

686 Bay Street, Toronto, ON, M5G 0A4, Canada

Shaun K. Morris ([shaun.morris@sickkids.ca](mailto:shaun.morris@sickkids.ca))

Division of Infectious Diseases, The Hospital for Sick Children

555 University Ave, Toronto, ON, M5G 1X8, Canada

**Journal Requirements:**

1. You indicated that you had ethical approval for your study. In your Methods section, please ensure you have also stated whether you obtained consent from parents or guardians of the minors included in the study or whether the research ethics committee or IRB specifically waived the need for their consent.

Author reply: We have clarified in our “Ethics statement” subsection (lines 124–125) that in households which contained minors, the consenting adult participant was always a parent, guardian, or other responsible primary caregiver of the minor (i.e., a grandparent).

2. In the ethics statement in the Methods, you have specified that verbal consent was obtained. Please provide additional details regarding how this consent was documented and witnessed, and state whether this was approved by the IRB.

Author reply: During data collection visits, trained data collectors first introduced themselves to an adult representative of the household and indicated that their household had been randomly selected to participate in a questionnaire. Data collectors described the information which would be collected including details of reproductive history, newborn health, and migration patterns for all members of the household; they also indicated participation was completely voluntary with no penalties for refusal. Eligible participants were encouraged to ask any questions about the survey including its aims, confidentiality regarding information to be collected, or why their household was selected.

The data collector then read a script directly from the questionnaire, which reads “WE ARE CONDUCTING A SURVEY OF HOUSEHOLDS IN THE COMMUNITY TO LEARN ABOUT THE REPRODUCTIVE HISTORY, NEWBORN HEALTH, MIGRATION PATTERNS, AND TELEPHONE AVAILABILITY IN SOME PARTS OF GB. THE INTERVIEW WILL TAKE ABOUT 15 MINUTES TO COMPLETE. ALL THE INFORMATION WE OBTAIN WILL REMAIN STRICTLY CONFIDENTIAL AND WILL BE USED TO INFORM FUTURE RESEARCH. MAY I START THE INTERVIEW?”. The data collector then documented the response on the physical questionnaire as either “Yes, permission given” or “No, permission not given”. The data collector also recorded their own name and data collector ID number on the questionnaire, prior to reading the consent script. Household members witnessed the data collector record this response.

The questionnaire was written in Urdu, the primary language of Gilgit-Baltistan. However, the consent discussion (and data collection for consenting participants) was conducted in the preferred language of the household. Data collection teams were comprised of four individuals, at least one of whom spoke the local language of the respective village, including Urdu, Balti (spoken in Baltistan), Shina (spoken in Astore), or Brushiski (spoken in Nagar). We have revised the “Ethics statement” subsection to reflect details of consent documentation and witnessing (lines 116–126), as well as language of the data collection procedures (lines 95–98).

3. Please include a complete copy of PLOS’ questionnaire on inclusivity in global research in your revised manuscript. Our policy for research in this area aims to improve transparency in the reporting of research performed outside of researchers’ own country or community. The policy applies to researchers who have travelled to a different country to conduct research, research with Indigenous populations or their lands, and research on cultural artefacts. The questionnaire can also be requested at the journal’s discretion for any other submissions, even if these conditions are not met.  Please find more information on the policy and a link to download a blank copy of the questionnaire here: [https://journals.plos.org/globalpublichealth/s/best-practices-in-research-reporting](https://can01.safelinks.protection.outlook.com/?url=https%3A%2F%2Fjournals.plos.org%2Fglobalpublichealth%2Fs%2Fbest-practices-in-research-reporting&data=05%7C01%7Cdaniel.farrar%40sickkids.ca%7C48ef56d9177f40666e1108dbcfe232ab%7C3961553ff47e49eb9f6ccf8518914e9a%7C0%7C0%7C638332342632709312%7CUnknown%7CTWFpbGZsb3d8eyJWIjoiMC4wLjAwMDAiLCJQIjoiV2luMzIiLCJBTiI6Ik1haWwiLCJXVCI6Mn0%3D%7C3000%7C%7C%7C&sdata=p7rb7P5yZoBFPvVnA33FGzwxZPMYFpPILmmRz4wILVs%3D&reserved=0). Please upload a completed version of your questionnaire as Supporting Information when you resubmit your manuscript.”

Author reply: We have included a copy of the *Inclusivity in global research* checklist as part of the submission of our revised manuscript.

4. Please review your reference list to ensure that it is complete and correct. If you have cited papers that have been retracted, please include the rationale for doing so in the manuscript text, or remove these references and replace them with relevant current references. Any changes to the reference list should be mentioned in the rebuttal letter that accompanies your revised manuscript. If you need to cite a retracted article, indicate the article’s retracted status in the References list and also include a citation and full reference for the retraction notice.

Author reply: We have reviewed the study reference list, and to our knowledge have referenced no retracted works. We note that reference #13 (Abu Fadaleh et al.) is a preprint by our team available on ResearchSquare which is currently under peer review. If the present manuscript is accepted at PLOS Global Public Health, we may be able to provide an updated reference #13 at a later date.

**Reviewer’s comments:**

1. This is a very well written manuscript, enriched with critically important data. I have only one comment: A big proportion of women who became pregnant and delivered during the pandemic period - might not have ANC PNC services which could have impact on the pregnancy outcome and thereby might impact on the observed result. This can be reported in the manuscript as one limitation aspect.

Author reply: We thank the reviewer for this comment, and agree that antenatal and perinatal care services were likely impacted between 2020–2021. In Gilgit-Baltistan, COVID-19-related mobility restriction policies were imposed which reduced interprovincial and inter-district travel, created capacity limits for various institutions, and separately, healthcare demands rose. We are unable to comment on how this may have affected most of our study outcomes, though do note the proportion of live births delivered at health facilities increased in 2020 relative to previous years (70.9%) relative to prior years (64.5% in 2017, 67.7% in 2018, and 68.9% in 2019; 2016 and 2021 are excluded as we did not ascertain the complete year in this study).

We have added the following text to the study limitations section (lines 323–327): “Finally, the study recall period included the first 1.5 years of the COVID-19 pandemic, a period when antenatal and perinatal care services may have been less commonly available and when an unknown proportion of the population were infected with SARS-CoV-2. We are unable to comment on what degree these factors affected the maternal and newborn outcomes presented in this study.”
